# Flavonoid Metabolites in Serum and Urine after the Ingestion of Selected Tropical Fruits

**DOI:** 10.3390/nu16010161

**Published:** 2024-01-04

**Authors:** Lalita Chomphen, Paveena Yamanont, Noppawan Phumala Morales

**Affiliations:** 1 Department of Pharmacology, Faculty of Science, Mahidol University, Bangkok 10400, Thailand; lalita_c@tistr.or.th (L.C.); paveena.cho@mahidol.ac.th (P.Y.); 2 Thailand Institute of Scientific and Technological Research, Pathum Thani 12120, Thailand

**Keywords:** bioavailability, flavonoids, kaempferol, luteolin, myricetin, quercetin, conjugated metabolites

## Abstract

The serum concentration and urinary excretion of flavonoids after the ingestion of guava, pineapple, and pomelo were determined using liquid chromatography–mass spectroscopy (LC-MS/MS). Each group of healthy volunteers was given 200 g of fresh fruit after overnight fasting and a 24-h flavonoid-free diet. The results demonstrate that only the glucuronic-conjugated metabolites of luteolin, quercetin, kaempferol, and myricetin were detected after fruit ingestion. The metabolites were first detected after 2 h, with the time to maximum concentration (T_max_) at 6 h. The most abundant metabolites for guava, pineapple, and pomelo were the glucuronide metabolites of quercetin (AUC_0–8_ 5.4 ± 1.3 μg·h/mL), kaempferol (AUC_0–8_ 9.9 ± 2.3 μg·h/mL), and luteolin (AUC_0–8_ 6.4 ± 1.1 μg·h/mL), respectively. The flavonoids found in the 24-h urinary excretions were glucuronic- and mainly sulfate-conjugated metabolites. Quercetin metabolites were the most abundant after guava and pineapple ingestion, accounting for 900 and 700 μg, respectively. Luteolin metabolites were the most abundant after pomelo ingestion, accounting for 450 μg. The serum and urinary metabolite profiles suggested that guava and pineapple are good sources of quercetin, pineapple is a good source of kaempferol, and pomelo is a good source of luteolin. The study of flavonoid profiles may provide information for the selection of fruits as functional foods for their health benefits to help with various health conditions.

## 1. Introduction

Flavonoids are one of the most important bioactive compounds present in vegetables and fruits. They exhibit a broad range biological activities and potency, prominently anti-oxidant [1] and anti-inflammatory [2]. The numerous epidemiology and meta-analysis studies support that a flavonoid-rich diet is associated with the reduced risk of non-communicable diseases such as cardiovascular diseases [3], diabetes [4], stroke [5,6], and cancer [7], and a lower risk of mortality in Parkinson patients [8].

Although flavonoids are the most common bioactive compounds found in plant-based diets, the low bioavailability, extensive metabolism, and rapid excretion of flavonoids may limit their biological activities in vivo [9]. The biological potency of metabolites recovered in blood and target organs may differ from the native substances. Therefore, the knowledge of the bioavailability and metabolites of dietary flavonoids is essential for selecting the appropriate fruits and calculating their portions as fruit-based functional food for health promotion and disease prevention.

Tropical fruits are a rich source of flavonoids. The most ubiquitous flavonoids in fruits are flavonols, including kaempferol, quercetin, and myricetin, whereas flavones, such as apigenin and luteolin, are less common [10]. We are interested in three kinds of tropical fruits, guava (*Psidium guajava* Linn.), pineapple (*Ananas comosus*), and pomelo (*Citrus grandis*), because of their high flavonoid content, taste, inexpensiveness, and common availability all year round in Thailand. 

Guava is one of the most popular fruits around the world. Pan-see-thong is a high-demand variety in the market. The fruit is round and has thin and light green skin. The edible mesocarp is thick with a creamy-white color. It is juicy and flavorful with a mild acidic and sweet taste. The center of the fruit is a cluster of small, hard, and flattened seeds about the size of a peppercorn. The medicinal uses of guava are common for diarrhea and dysentery, controlling weight and blood glucose levels [11]. 

Pomelo is the largest citrus fruit, native to South and Southeast Asia. Thong-dee is the predominant commercial variety in Thailand. The fruit is oblate with approximately 1 kg in weight. It has a very thick and soft green peel. The flesh is pinkish at about 1 cm from the outside. The fruit has juice sacs with a sweet to slight sour taste. The health benefits of pomelo are associated with its anti-oxidant activity of bioactive compounds such as polyphenols and flavonoids [12]. 

Pineapple is a perennial monocot of the family Bromeliaceae. Phuket is in the group of Queen pineapples. It has a small cylindrical shape with brownish yellow skin, dry, bright yellow flesh, crispy, sweet taste, and a delicate mild flavor. Consumption of pineapple helps to boost the immune system. Moreover, bromelain is a bioactive compound that aids digestion and exhibits anti-inflammatory and analgesic properties [13].

This study aims to determine the serum concentration of flavonoids and metabolites, as well as the urinary excretion of flavonoids in healthy volunteers after the ingestion of the three selected tropical fruits. The fruit varieties with the highest content of flavonoids were selected in this study. Pan-see-thong is a guava variety that has a total flavonoid content of about 8.93 mg per 100 g edible portion. Thong-dee, a variety of pomelo, and Phuket, a variety of pineapple, have a total flavonoid content of 13.9 and 4.26 mg per 100 g edible portion, respectively. The most abundant flavonoids in guava, pomelo, and pineapple are quercetin, luteolin, and kaempferol, respectively [10]. The results of metabolize in blood and urine may be useful for the selection of Thai fruits as a functional food to help manage specific health conditions.

## 2. Materials and Methods

### 2.1. Fruit Samples

Guava (Pan-see-thong), pineapple (Phuket), and pomelo (Thong-dee) were obtained from Simummuang market, Pathum Thani, Thailand. All fruit samples were washed several times with tap water, peeled, and rinsed again with deionized water to ensure that all contaminations and peels were removed. A serving size (200 g) of edible parts was given to the participants. 

### 2.2. Participants 

Three groups of six healthy subjects (male and female) between 20 and 30 years of age, with a body mass index between 18 and 25, were recruited for this study. Each group of participant received one kind of fruit. They were not alcohol drinkers, smokers, drug users, nor pregnant. They had not taken any medications for at least one month prior to the experiment. All participants underwent health checks for their hematological and biochemical profiles. The data are provided in Supplementary Table S1. There was no clinical presentation of gastrointestinal diseases (peptic ulcer), kidney diseases, liver diseases or jaundice, heart diseases, asthma, or diabetes mellitus. Levels of blood glucose before and 4 h after fruit ingestion were monitored in all subjects. The results are shown in Supplementary Table S2. The study protocol was submitted for approval by the Ethical Committee on Human Rights of the Faculty of Medicine, Ramathibodi Hospital, Mahidol University (Protocol code. 08-54-18). Informed consent was obtained from all subjects involved in the study.

### 2.3. Study Design 

Participants maintained a low-flavonoid diet (e.g., diet without vegetables, fruits, or chocolate) for two days and fasted for 8 h before the study to avoid any contribution from other foods containing flavonoids. Servings of fresh fruits of 200 g were given to the participants followed by 200 mL water. Beverages containing alcohol and caffeine were prohibited during the test period. Venous blood samples were collected before and 2, 4, 6, and 8 h after fruit ingestion. Serum was immediately prepared using centrifugation 233× *g* at 4 °C for 15 min, 200 mM ascorbic acid was added, and the samples were then stored at −20 °C until the analysis (within two weeks). Urine samples were collected at intervals of 0–4, 4–6, 6–8, 8–12, and 12–24 h in plastic bottles containing 70 mg of ascorbic acid per 100 mL of urine. The volume of urine sample was measured and then centrifuged at 233× *g* to remove the precipitate. Ten milliliters of each sample were collected for freeze-drying (Labconco, Kansas City, MO, USA) and stored at −20 °C until the analysis. Participants had lunch and dinner, which did not contain fruits and vegetables, at 4 and 12 h after the ingestion of fruits.

### 2.4. Sample Preparation

The serum and freeze-dried powder of urine samples were diluted with 2 and 3 mL of 0.5 M phosphoric acid, respectively, before solid-phase extraction using an OasisTM HLB cartridge (30 mg; Milford, MA, USA) [14]. The cartridge was placed on a vacuum manifold and conditioned by wetting it with 1 mL of 0.5 M phosphoric acid solution before loading the sample. The cartridge was first washed with 1 mL of 5% methanol in 0.5 M phosphoric acid solution, followed by 1 mL of 50% methanol in 0.5 M phosphoric acid solution and 3 mL of methanol. The eluted samples were filtered through 0.20 µm cellulose membrane (13 mm, syringe filter, CA 0.2 µm, Thermo Fisher Scientific, Waltham, MA, USA). Methanol was removed using a speed vacuum concentrator (SAVANT Model SC210A, Thermo electron Corporation, Waltham, MA, USA). The residue was re-dissolved with 200 µL of mobile phase solution and used for the determination of flavonoids.

The conjugated metabolites of flavonoids, glucuronic acid, and sulfate conjugates were determined in the serum and urine after hydrolyzing with β-glucuronidase (EC 3.2.1.31, bovine liver type B-I, Sigma, St Louis, MO, USA) and aryl sulfatase (EC 3.1.6.1, Merk, Darmstadt, Germany), respectively, at 37 °C for 16 h. After hydrolysis, the serum and urine samples were processed as described above. 

### 2.5. LC/ESI-MS/MS Analysis 

Flavonoid analysis was measured by a modified method of Biessaga [15] and Mullen et al. [16]. This system was carried out using Hypersil BDS C18 column (250 mm × 4.6 mm I.D., 5 μm, Thermo Scientific, Waltham, MA, USA). The mobile phase was composed of 0.1% formic acid in water (A) and 0.1% formic acid in acetonitrile (B). Separations were affected by a gradient using a flow rate of 0.2 mL/min as follows: elution started with 70% A, 5–10 min; 60% A, 15–17 min; 50% A, 19 min; 60% A, 25–45 min; 70% A. A column eluent was directed into an ESI/ion trap mass spectrometer (Agilent 1100 series, Waldbornn, Germany). The mass was run in positive ion mode using a capillary voltage of 25 eV and capillary temperature of 350 °C. Absorbance was recorded using a PDA (Bruker esquire HCT series, Bruker, Bremen, Germany) at the wavelength of 200–400 nm. Peak areas were integrated with Bruker Daltonics Data Analysis Version 3.4 (Bruker, Germany). 

In the present work, luteolin, kaempferol, quercetin, and myricetin showed the single-charged molecular ion [M + H]^+^ with *m*/*z* 287, 287, 303, and 319, respectively. The collision-induced dissociation technique was used to confirm the chemical structure of the flavonoids by analyzing the product mass after applying the appropriate fragmentation energy (1.20–1.35 volts). The most abundant products were *m*/*z* 258, 241, 257, and 273 for luteolin, kaempferol, quercetin, and myricetin, respectively. Flavonoids were identified by comparison retention time (RT) and *m*/*z* values obtained from MS and MS2 with standard flavonoids. The flavonoid concentrations were calculated from the calibration curve obtained from the abundant product mass in multiple reaction monitoring (MRM) mode. Mass spectra and mass chromatograms of the flavonoids and the ions they produced after fragmentation are shown in Supplementary Figures S1 and S2.

### 2.6. Calibration Curve and Method Validation

To construct calibration curves, a mixed standard solution of luteolin, kaempferol, quercetin, and myricetin (Sigma, St. Louise, MO, USA) was spiked in the pooled samples of five blank serum or urine concentrations (10–400 ng/mL) extracted and analyzed according to the above-described methods. The concentrations of flavonoids in serum and urine were calculated using calibration curves.

The linearity of the assay was over the concentration range of 20–400 ng/mL with the coefficient of determination (r^2^) more than 0.99. The detection limits were determined based on comparing the analyzed peak height obtained from the spiked sample with the three times height of baseline signal. The value of the lower limit of the quantization (LLOQ) of flavonoids in serum and urine was 20 ng/mL. The percentages of recovery over the concentration range 20–400 ng/mL were between 83.8 and 107.0 for flavonoids that spiked in serum analyses, and between 72.1 and 106 for flavonoids that spiked in urine analyses. The analysis of the inter-day and intra-day precision and accuracy of flavonoids were in the acceptable range. 

### 2.7. Data and Statistical Analysis 

The serum concentration–time curve of individual subjects was constructed to determine the maximum concentration (C_max_) and time to reach C_max_ (T_max_). The area under the concentration–time curve from time zero to eight hours (AUC_0–8_) was calculated using the trapezoidal rule. Concentrations of conjugated metabolites of flavonoids were presented as free flavonoid equivalence. All data were presented as mean ± SD, unless otherwise indicated. 

## 3. Results

### 3.1. Mass Chromatograms and Flavonoid Profiles in Serum

**Figure 1:**
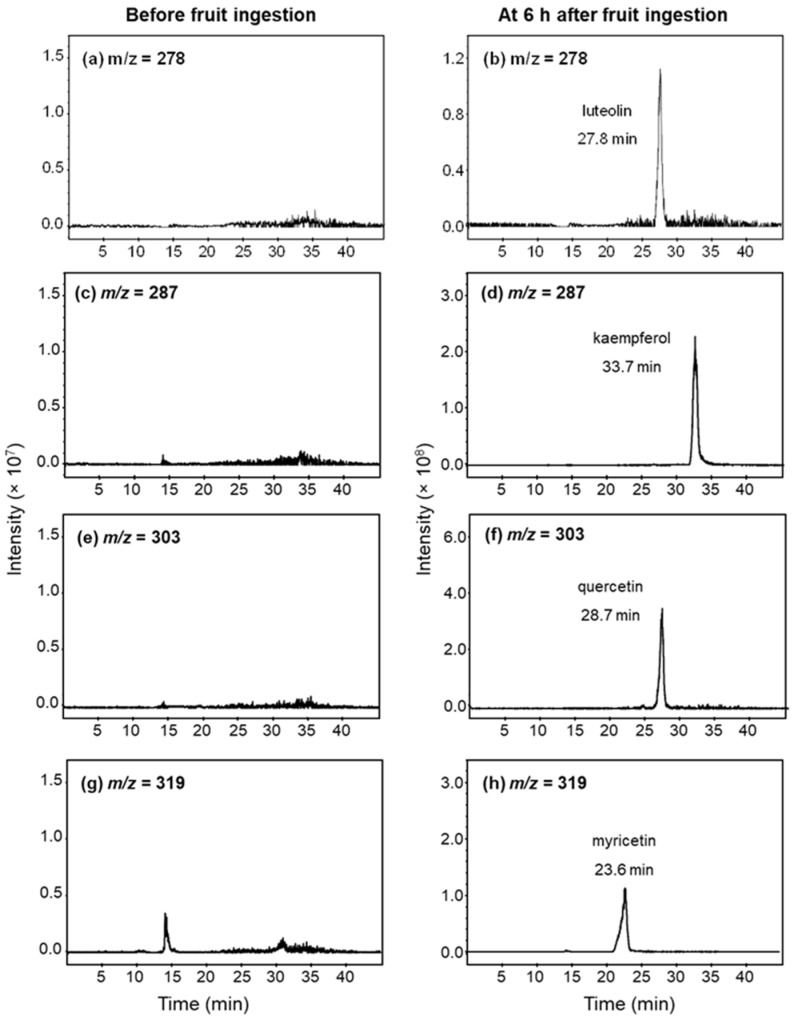
Example of mass chromatograms of luteolin (*m*/*z* = 278), kaempferol (*m*/*z* = 278), quercetin (*m*/*z* = 303), and myricetin (*m*/*z* = 319) in serum after hydrolysis with β-glucuronidase. Serum samples were obtained before (left panel) and 6 h (right panel) after ingestion of 200 g of (**a**,**b**) guava; and (**c**–**h**) pineapple.

The examples of mass chromatograms in serum before and after fruit ingestion are shown in Figure 1. There was no detectable amount of flavonoids in the serum of all participants after being on a low-flavonoid diet for 48 h prior to the study. All flavonoids in the serum were detected as glucuronic-conjugated metabolites, but none were detected as sulfate metabolites or free-form flavonoids. The chromatograms of flavonoids after hydrolysis with β-glucuronidase are demonstrated in Figure 1.

**Figure 2:**
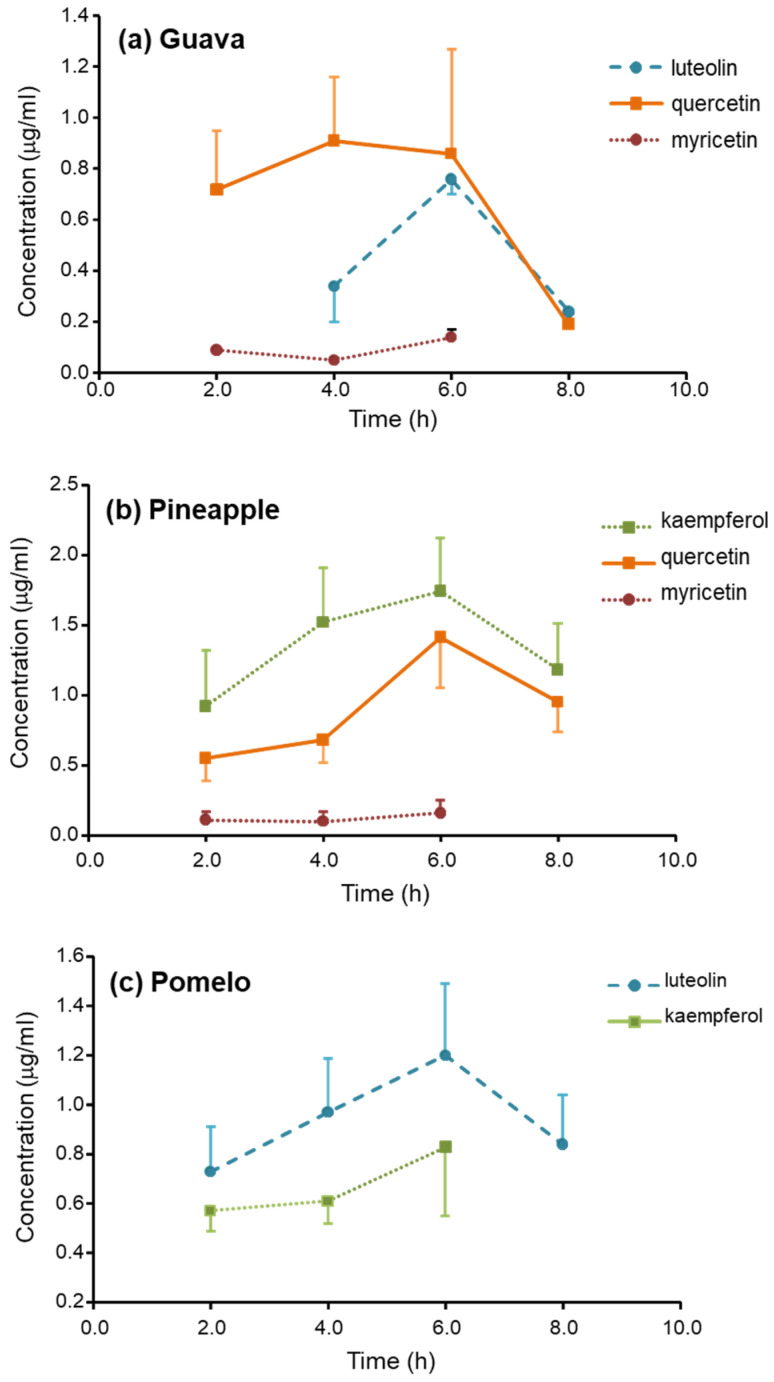
Serum concentration-time profiles of flavonoids after ingestion of 200 g (**a**) guava; (**b**) pineapple; and (**c**) pomelo.

There are only four flavonoids detected in the serum: luteolin (*m*/*z* = 287, retention time (RT) = 27.8), quercetin (*m*/*z* = 303, RT = 28.7), and myricetin (*m*/*z* = 319, RT = 23.4) were found after guava ingestion; kaempferol (*m*/*z* = 287, RT = 33.7), quercetin, and myricetin were found after pineapple ingestion; and kaempferol and luteolin were found after pomelo ingestion. The serum profiles of flavonoid glucuronic-conjugated metabolites are shown in Figure 2. 

The T_max_ of the glucuronic-conjugated metabolites of all flavonoids was found at 6 h. The values of the C_max_ and the AUC_0–8_ of the metabolites are demonstrated in Table 1. The metabolites of quercetin were found as the most abundant after guava ingestion, with AUC_0–8_ was about 5.4 μg·h/mL. The metabolites of kaempferol and luteolin, with AUC_0–8_, were about 9.9 and 6.4 μg·h/mL and were the most abundant after the ingestion of guava and pineapple, respectively. The C_max_ of the most abundant metabolites was approximately ≥1 μg/mL. 

### 3.2. Flavonoid Excretion in Urine

**Figure 3:**
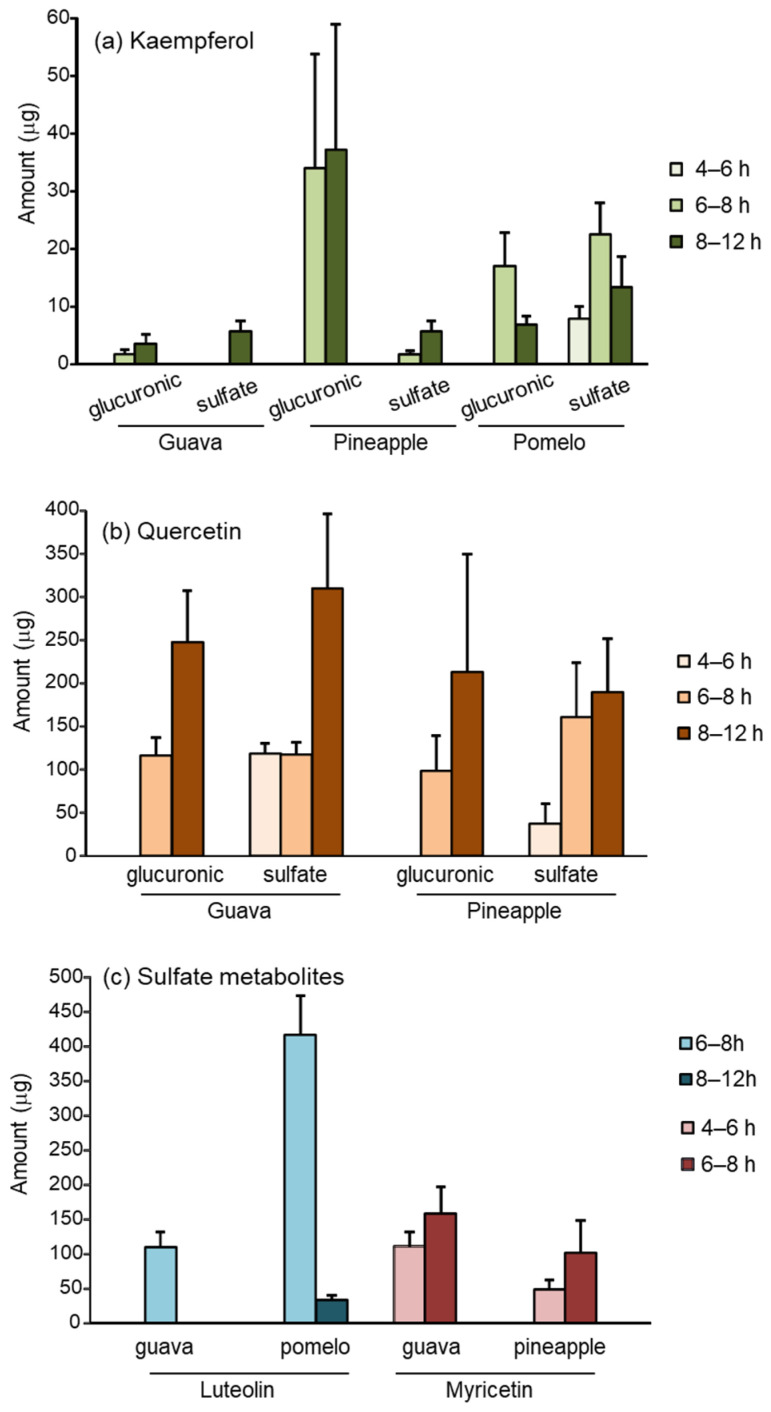
Urinary metabolites of (**a**) kaempferol; (**b**) quercetin; (**c**) luteolin, and myricetin recovered in various time intervals after ingestion of 200 g guava, pineapple, and pomelo.

In urine, both the glucuronic- and sulfate-conjugated metabolites of flavonoids were recovered. Figure 3 demonstrates the urinary excretion of flavonoid metabolites at various time intervals. The total urinary excretion is summarized in Table 2. The glucuronic metabolites were detected at 6–8 h, whereas sulfate metabolites were detected earlier at 4–6 h. There was no detectable flavonoid metabolite at 0–4 or 12–24 h after the ingestion of the fruits. Kaempferol and quercetin were found in both conjugated metabolites (Figure 3a,b), whereas luteolin and myricetin were only detected in sulfate form (Figure 3c). 

Similar to the levels found in serum, quercetin and luteolin were the most abundant flavonoids recovered in urine, accounting for 900 μg and 450 μg after the ingestion of guava and pomelo, respectively. The urinary excretion of quercetin and kaempferol was approximately 700 μg and 500 μg after pineapple ingestion.

## 4. Discussion

The metabolite profiles of flavonoids after the consumption of three tropical fruits (guava, pineapple, and pomelo) in the serum and urine of healthy participants were demonstrated. The conjugated metabolites of flavonols (kaempferol, quercetin, and myricetin) and flavone (luteolin) were detected in our study. Flavonols and flavone are metabolized by phase II enzymes in the small intestine and liver before reaching blood circulation. Therefore, unconjugated flavonoids were not detected in the circulation. The serum levels of glucuronide metabolites were detected within the first 2 h, developed peak serum concentration at 4 to 6 h, and lasted more than 8 h. 

The variation in the pharmacokinetics of flavonoids in different forms and food sources has been reported in humans [16,17,18,19,20]. A study by Graefe et al. [21] demonstrated that the T_max_ of quercetin was 0.7 h after a single ingestion of onion and quercetin-4’-glucoside. However, a longer T_max_ of 4.3 and 6.9 h was reported after ingesting buckwheat tea and quercetin-3-rutioside. Our results showed the comparable T_max_ of glucuronide metabolites of quercetin was obtained about 4 and 6 h after ingestion of guava and pineapple. The metabolites of luteolin were detected with a T_max_ of 6 h after ingestion of pomelo. In contrast, Breiter et al. reported a T_max_ of 1.5–3 h after ingestion of rooibos tea [22]. The variation in the absorption phase pharmacokinetics of flavonoids may be explained by their different forms and the interaction with insoluble fibers and other contents in the fruits [23]. 

The bioavailability of flavonoids is influenced by their forms, aglycones and glycosides. Aglycones are more readily absorbed in the small intestine, whereas glycosides are absorbed via sodium-dependent glucose transporter 1 (SGLT1) or metabolized by lactase phlorizin hydrolase (LPH) before absorption as aglycones. Furthermore, flavonoids extensively undergo glucuronidation with phase II metabolizing enzymes, uridine diphosphate glucuronosyl transferases (UDPGT), and future sulfation via sulfotransferase (SULT) in both enterocytes and hepatocytes before distribution to the bloodstream [24,25]. Our study showed that flavonols, particularly kaempferol and quercetin, were absorbed and readily metabolized into flavonoid glucuronides. Despite the presentation of apigenin and hesperetin in the selected fruits [10], we did not detect them in serum or urine. This may be explained by the limited bioavailability of apigenin [26,27] and hesperetin [28]. Moreover, an in vitro study demonstrated that apigenin undergoes phase I metabolism and forms luteolin as a major metabolite [29]. We presume that the detected luteolin in the circulation after guava and pomelo ingestion may partly derive from apigenin.

The small conjugates such as monosulfates are preferentially excreted in urine, whereas the larger conjugated metabolites are more likely to be eliminated in bile. Furthermore, glucuronic-conjugated metabolites also occur in entero-hepatic circulation [30]. Therefore, this may explain why only glucuronide metabolites were detected in serum, and its levels in serum lasted longer than 8 h due to enterohepatic circulation. Correspondingly, the sulfate conjugate metabolites rapidly recovered in urine during the first 4 h, while glucuronic-conjugated metabolites were detected 6 h after fruit ingestion. 

The sulfate conjugate metabolites accounted for more than 80% of the total urinary excretion of kaempferol, myricetin, and luteolin. However, the urinary glucuronide and sulfate metabolites of quercetin were relatively comparable. In general, sulfation is a higher affinity, lower capacity pathway than glucuronidation. As a result, the shift from sulfation toward glucuronidation occurs when the ingested dose increases [31]. We postulate that the metabolite profiles of dietary flavonoids may depend on the quality and quantity of flavonoids in the food sources. 

According to the serum and urinary excretion profiles, we propose that guava is a good source of quercetin and myricetin, pineapple is a good source of quercetin and kaempferol, and pomelo is a good source of luteolin. Besides the amount of flavonoids recovered in serum and urine, the bioavailability of flavonoids is a key factor in calculating a portion of fruit consumption. 

Several studies revealed that the forms and food sources of flavonoids have an impact on their bioavailability [16,17,18,19,20]. Hollman et al. [17] reported the bioavailability of quercetin from apples and pure quercetin-3-rutinose was only 30% compared to onions. Here, we assumed the flavonoids recovering in urine had passed the blood compartment. Hence, we estimated the bioavailability of flavonoids based on the total urinary excretion of flavonoids and flavonoid content in fruits reported by Kongkachichai et al. [10]. In the two-hundred grams of edible part, guava and pineapple contained about 6900 μg and 850 μg of quercetin, respectively. The total amount of urinary quercetin accounted for 13% and 80% of the quercetin content in the respective fruits. Hence, bioavailability kaempferol was 1% and 10% from pomelo and pineapple; myricetin was 5% and 10% from guava and pineapple; luteolin was 5% in both guava and pomelo. As result, we suggest that bioavailability of flavonoids from pineapple was higher than other selected fruits. It should be noted that, the bioavailability of flavonoids may be underestimated because the biliary excretion of flavonoids was not taken into account. Moreover, the urinary methyl-conjugated favonoids were not quantified in this study. 

Flavonoids have been recognized as potent antioxidants and possess several biological activities that benefit human health. Structure–activity relationship (SAR) studies indicate that the number and location of the phenolic hydroxyl groups are essential for antioxidant activity. The presence of a 3′,4′-dihydroxy group in the B ring and 3-hydroxyl moiety, as well as the C2–C3 double bond conjugate with a 4-keto group, are important for the radical scavenging activity of flavonoids [32,33].

Morand et al. [34] demonstrated the decreasing potency of the glucuronide and sulfate metabolites of quercetin against lipoprotein oxidation. However, the potency of the metabolites was about five times higher than tolox. In plasma, the major metabolites were present in mono-conjugated forms. About 20–40% of kaempferol, quercetin, and myricetin is glucuronide in the 3′-position, implying a decrease in the antioxidant capacity of dietary flavonoids in vivo [32]. Nevertheless, other contributions from essential structures, such as the 3-hydroxyl group, still maintain the antioxidant activity of flavonoids. The bioactivities of the conjugated metabolites of flavonoids have been extensively reviewed, especially the conjugated metabolites of quercetin [35,36].

The position of a conjugated substitute is also crucial for the biological activities and hydrophilicity of flavonoid metabolites [37]. The 4′- and 3′-quercetin glucuronides showed a potent inhibitory effect of xanthine oxidase, whereas the 3-glucuronide metabolite was not active [38]. Recently, Nishikawa et al. [39] demonstrated the biological properties of the positional isomers of quercetin mono-glucuronides in activated macrophages. Yu et al. [40] demonstrated that quercetin-3-glucuronide exerted antioxidant and anti-inflammatory activity in LPS-induced pulmonary injury in both in vitro and in vivo models. Żyżyńska-Granica et al. [41] showed that flavonoid aglycones, not their glucuronides, showed anti-inflammatory activity in PMNs and HUVECs. However, the β-glucuronidase release from inflammatory cells could deactivate the anti-inflammatory activity of flavonoid glucuronides. These studies support the therapeutic potential of dietary flavonoids and their metabolites. 

The limitation of this study is that we could not identify the metabolite profiles of flavonoids by analyzing the mass shift in the chromatograms [42]. Although the method of enzyme digestion with β-glucuronidase and sulfatase is suitable for quantitative analysis, it could not provide information on the number and position of conjugation in the molecules. This limitation may relate to the limit of quantification of our method that may not cover the low consumption amount of flavonoids compared with the previous studies or the low bioavailability of flavonoids. In our study, the approximate consumption amount of each flavonoid was less than 10 mg in a serving size, whereas a tenfold higher amount was given in humans in other reports [16,17,18,19,20,43]. 

## 5. Conclusions

In conclusion, the conjugated metabolites of kaempferol, quercetin, myricetin, and luteolin were recovered in serum and urine after the consumption of the selected tropical fruits. Quercetin in guava and pineapple showed a higher bioavailability than the other detected flavonoids, suggesting that these fruits are good sources of quercetin. The study of the metabolite profiles of flavonoids in humans may help to select the appropriate food sources for flavonoids to obtain health benefits. Since the biological activities of the conjugated metabolites of flavonoids have been reported, the accumulation of the conjugated metabolites in the blood could produce health benefits after fruit intake.

## Figures and Tables

**Table 1:** Serum concentration of glucuronic-conjugated metabolite of flavonoids after ingestion of various fruits.

		Parameter	
Fruit Ingestion	Flavonoid	C_max_ (μg/mL)	AUC_0–8_ (μg·h/mL)
Guava	Luteolin	0.8 ± 0.1	2.3 ± 0.9
	Quercetin	1.1 ± 0.2	5.4 ± 1.3
	Myricetin	0.14 ± 0.03	0.6 ± 0.5
Pineapple	Kaempferol	1.7 ± 0.4	9.9 ± 2.3
	Quercetin	1.4 ± 0.4	6.5 ± 1.5
	Myricetin	0.2 ± 0.1	0.8 ± 0.3
Pomelo	Luteolin	1.2 ± 0.3	6.4 ± 1.1
	Kaempferol	0.8 ± 0.3	3.2 ± 1.0

Data are presented as mean ± SD (*n* = 6). Concentrations of flavonoid metabolites are presented as μg of free-flavonoid equivalence. C_max_ = maximum serum concentration; AUC_0–8_ = area under the serum concentration–time curve from time 0 to 8 h.

**Table 2:** Twenty-four hours urinary excretion of flavonoids excretion as glucuronic and sulfate conjugated metabolites.

		Amount (μg)		
Fruit Ingestion	Flavonoid	Glucuronide	Sulfate	Total
Guava	Luteolin	-	109.5 ± 22.0	109.5 ± 22.0
	Kaempferol	5.2 ± 1.7	7.4 ± 2.0	12.6 ± 3.5
	Quercetin	363.8 ± 68.6	546.2 ± 78.4	910.0 ± 137.3
	Myricetin	-	269.8 ± 33.4	269.8 ± 33.4
Pineapple	Kaempferol	71.2 ± 40.9	419.1 ± 135.4	490.3 ± 161.3
	Quercetin	311.6 ± 171.4	387.8 ± 122.8	699.4 ± 28.4
	Myricetin	-	149.6 ± 51.8	149.6 ± 51.8
Pomelo	Luteolin	-	450.3 ± 55.9	450.3 ± 55.9
	Kaempferol	23.8 ± 4.8	43.6 ± 5.2	67.0 ± 8.9

Data are presented as mean ± SD (*n* = 6). The amount of flavonoid metabolites is presented as free-flavonoid equivalence.

## Data Availability

All data are contained within the article.

## Supplementary Materials

The following supporting information can be downloaded at: https://www.mdpi.com/article/10.3390/nu16010161/s1, Figure S1: mass spectrum of flavonoids and product ions after fragmentation; Figure S2: mass chromatogram of flavonoids and their product ions after fragmentation; Table S1: demographic, biochemical, and hematological profile of participants; Table S2: levels of blood glucose before and 4 h after fruit ingestion.
