# Supplementary material for: Flavonoid Metabolites in Serum and Urine after the Ingestion of Selected Tropical Fruits
Source: Nutrients. 2024 Jan 4;16(1):161. doi: 10.3390/nu16010161 (PMC10780413; doi:10.3390/nu16010161)
Supplement: Supplementary file 1 [file nutrients-16-00161-s001.zip › nutrients-2770919-supplementary.pdf]

Supplementary Data

Table S1 Demographic, biochemical and hematological profile of participants

**Parameter**	**Group I (Pineapple)**	**Group II (Guava)**	**Group III (Pomelo)**
n	6	6	6
Gender (M/F)	6/0	5/1	5/1
Age (years)	24.5 (21-29)	25 (21-29)	24.5 (21-29)
BMI (kg/m^2)	22.1 (20.7-23.1)	22.0 (20.9-23.1)	22.0 (20.5-23.1)
ALP (U/L)	88 (79-100)	88 (71-98)	90 (71-105)
AST (U/L)	16.5 (12-22)	21.5 (16-27)	21 (15-27)
ALT (U/L)	35 (22-44)	36 (34-56)	37.5 (32-56)
GGT (U/L)	31 (38-41)	35 (25-40)	36 (25-41)
Albumin (g/L)	43.15 (41.1-44.3)	45.75 (40-47.8)	43.1 (40-47.7)
BUN (mg/dL)	9.5 (7-11)	12.5 (11-17)	12.5 (9-17)
Creatinine (mg/dL)	0.78 (0.64-0.99)	0.83 (0.8-1.07)	0.83 (0.7-1.07)
Triglyceride (mg/dL)	70.5 (51-96)	81 (48-147)	64.5 (48-137)
Cholesterol (mg/dL)	189 (153-201)	199 (159-216)	198 (180-213)
Total bilirubin (mg/dL)	0.5 (0.4-0.7)	0.5 (0.2-0.7)	0.6 (0.2-0.9)
WBC (x 10^3 /μL)	5.9 (5.4-6.9)	6.4 (4.7-8.9)	6.7 (4.7-8.9)
RBC (x 10^6 /μL)	5.3 (4.8-6.0)	5.2 (4.6-5.9)	5.3 (4.6-5.6)
Hb (g/dL)	14.2 (12.3-16.6)	14.4 (12.4-16.0)	14.4 (12.3-16.0)
HCT (%)	42.8 (36.7-49.8)	44.0 (37.7-48.8)	44.0 (37.7-48.3)
MCV (fL)	86.4 (80.6-89.8)	85.5 (81.6-9.12)	86.7 (81.6-89.8)
MCHC (g/dL)	33.4 (33.3-34.6)	34.1 (33.0-34.9)	34.2 (33.0-34.9)
PLT (x 10^3 /μL)	268 (223-312)	268 (218-384)	334 (264-384)
MPV (fL)	10.0 (9.5-11.0)	9.20 (8.6-10.0)	9.05 (8.5-10.0)

Data are presented as median (Min-Max)

APL, alkaline phosphatase; AST, aspartate aminotransferase; ALT, alanine aminotransferase; GGT, gramma-glutamyltranpeptidase; BUN, blood urea nitrogen; WBC, white blood cell count; RBC, red blood cell count; Hb, hemoglobin, HCT, hematocrit; MCV, mean corpuscular volume; MCHC, mean corpuscular hemoglobin concentration

Table S2 Levels of blood glucose before and 4 hours after fruits ingestion

Group	Blood glucose levels (mg/dL)	
	**Before ingestion**	**4 Hours after ingestion**
Group I (Pineapple)	90.5 (84-97)	86.5 (77-98)
Group II (Guava)	79.5 (73-86)	78.5 (72-84)
Group III (Pomelo)	79.5 (73-97)	76.5 (70-79)

Data are presented as median (Min-Max). Normal blood glucose level is 70-110 mg/dL.

Figure S1 Mass spectrum of flavonoids and product ions after fragmentation

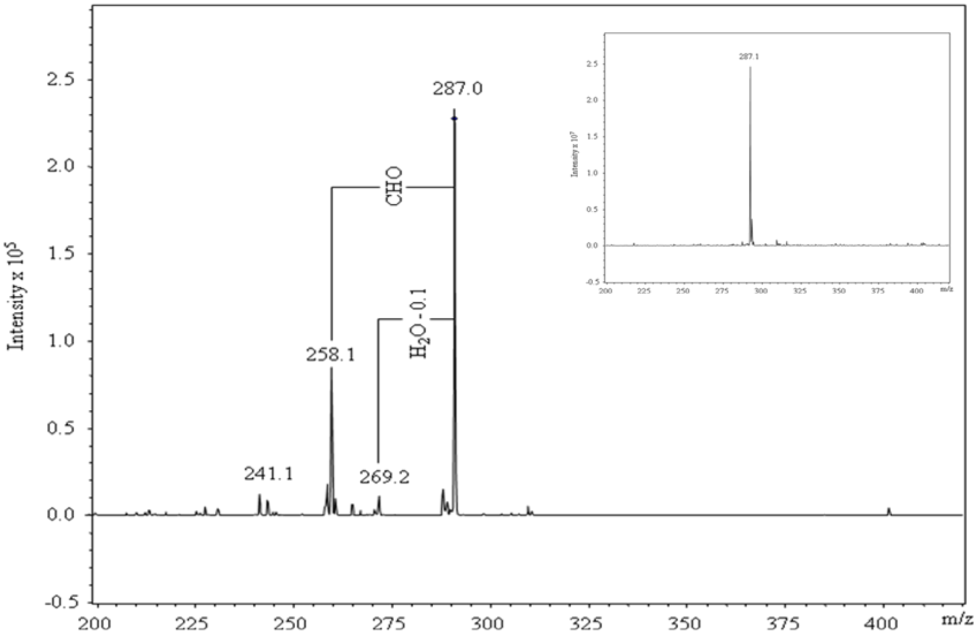

Figure S1.1 Mass spectrum of **luteolin**, m/z 287 $[M+H]^+$ by direct infusion of standard solution (insert) and product ions after fragmentation by applied energy 1.20 volts at m/z 287

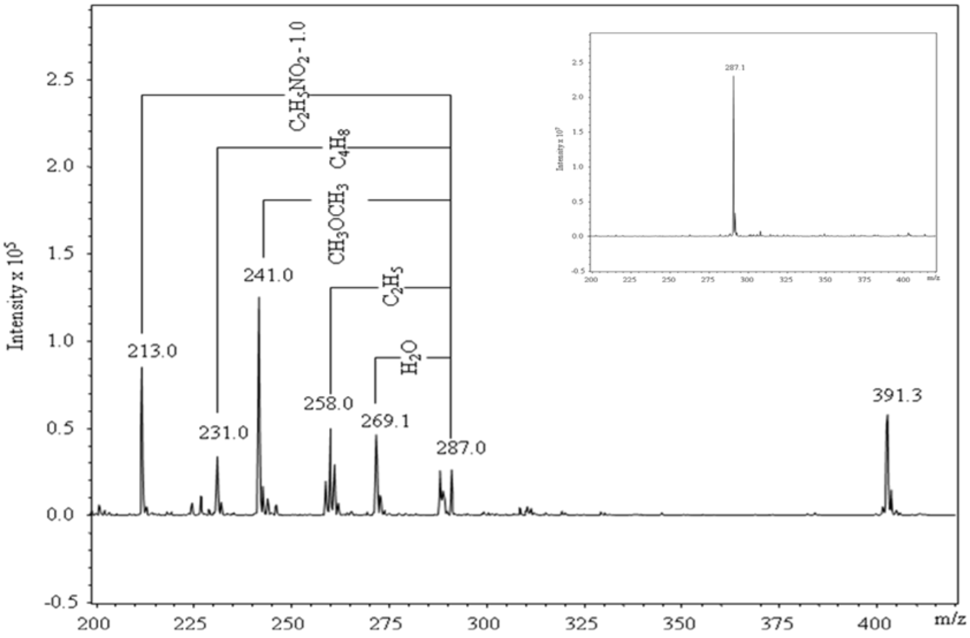

Figure S1.2 Mass spectrum of **kaempferol**, m/z 287 $[M+H]^+$ by direct infusion of standard solution (insert) and product ions after fragmentation by applied energy 1.30 volts at m/z 287

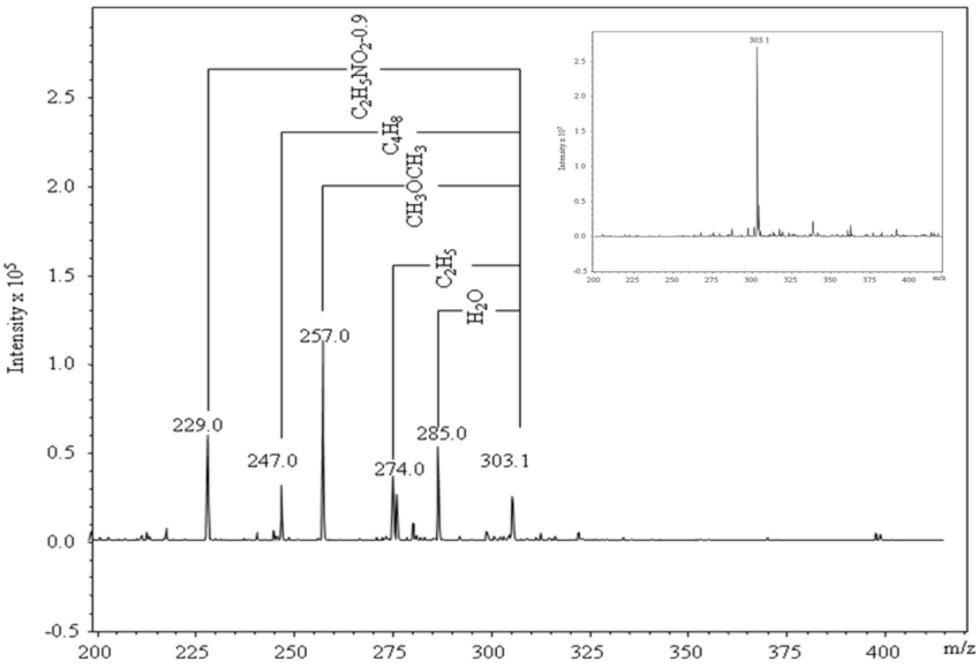

Figure S1.3 Mass spectrum of **quercetin**, m/z 303 $[M+H]^+$ by direct infusion of standard solution (insert) and product ions after fragmentation by applied energy 1.35 volts at m/z 303

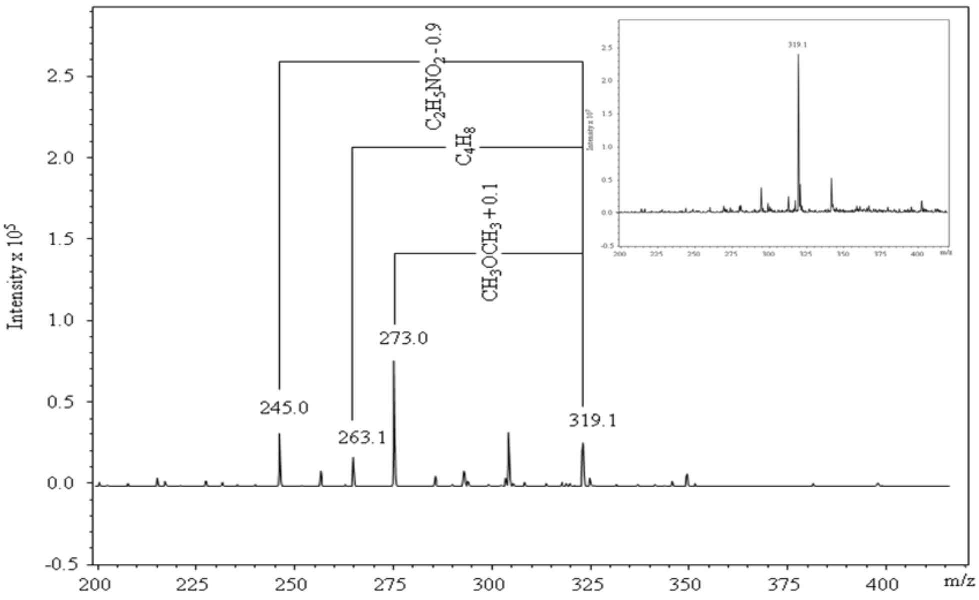

Figure S1.4 Mass spectrum of **myricetin**, m/z 319 $[M+H]^+$ by direct infusion of standard solution (insert) and product ions after fragmentation by applied energy 1.30 volts at m/z 319.

Figure S2 Mass chromatogram of flavonoids and their product ions after fragmentation

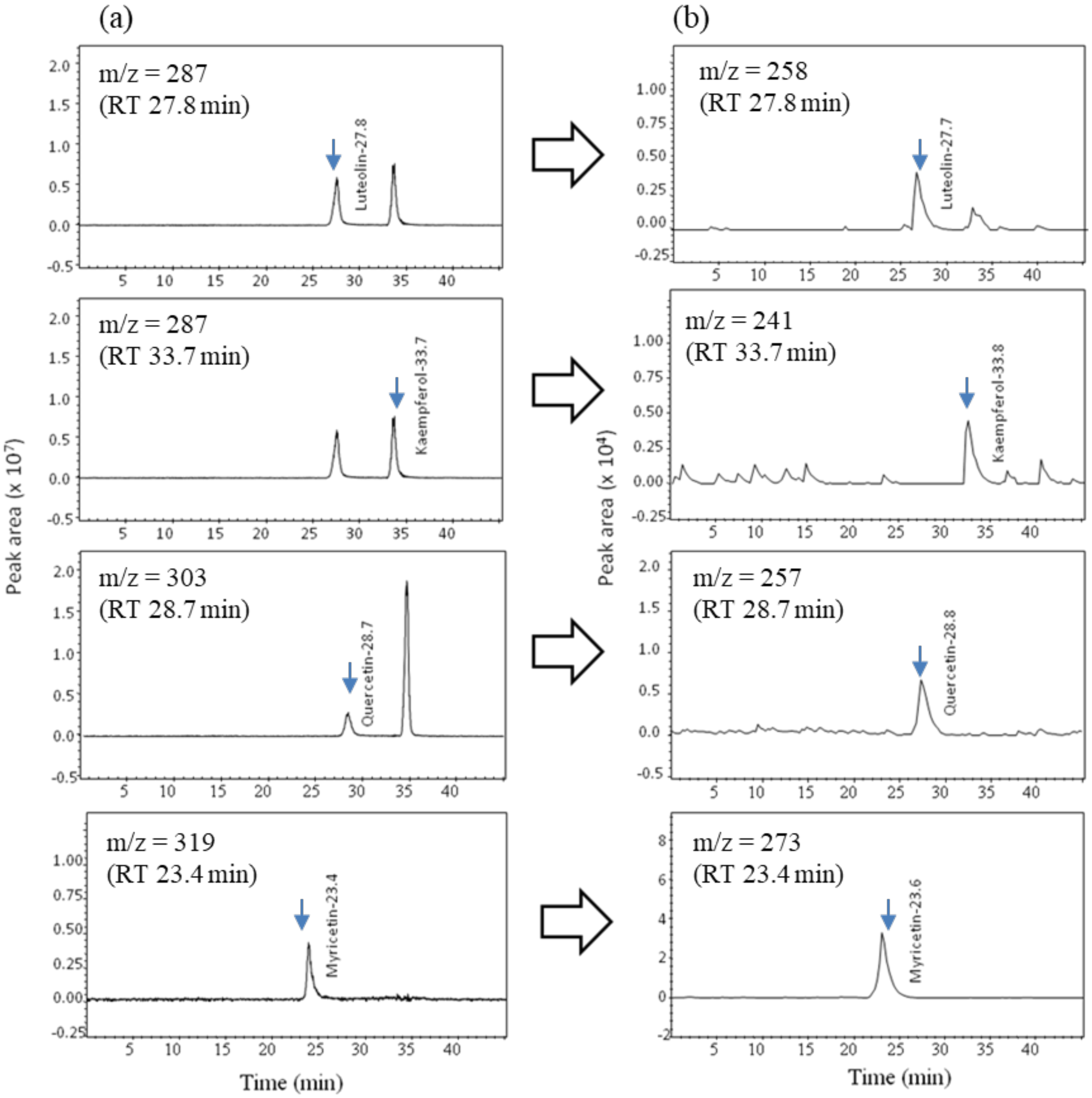

Figure S2 Mass chromatogram of luteolin (m/z = 287), kaempferol (m/z = 287), quercetin (m/z = 303), and myricetin (m/z = 319) at concentration 1 µg/mL (a), and their respective product ions after fragmentation (b).
